# Transcriptional landscape of mouse-aged ovaries reveals a unique set of non-coding RNAs associated with physiological and environmental ovarian dysfunctions

**DOI:** 10.1038/s41420-018-0121-y

**Published:** 2018-12-05

**Authors:** Danila Cuomo, Immacolata Porreca, Michele Ceccarelli, David W. Threadgill, William T. Barrington, Annacristina Petriella, Fulvio D’Angelo, Gilda Cobellis, Francesca De Stefano, Maria N. D’Agostino, Mario De Felice, Massimo Mallardo, Concetta Ambrosino

**Affiliations:** 1 Department of Science and Technology, University of Sannio, Via Port’Arsa 11, 82100 Benevento, Italy; 2 Department of Molecular and Cellular Medicine, College of Medicine, Texas A&M University, College Station, TX 77843 USA; 3 IRGS, Biogem, Camporeale, 83031 Ariano Irpino, Avellino Italy; 4 Department of Veterinary Pathobiology, College of Veterinary Medicine & Biomedical Sciences, Texas A&M University, College Station, TX 77843 USA; 5 Department of Experimental Medicine, Second University of Naples, Via Costantinopoli 16, 80138 Naples, Italy; 6 Department of Children and Women Health, Physiopathology of Human Reproduction Unit, A.O.R.N. S.G. Moscati, 83100 Avellino, Italy; 7 Molecular Medicine and Medical Biotechnologies, University of Naples “Federico II”, 80131 Naples, Italy; 8 IEOS-CNR, Via Pansini 6, 80131 Naples, Italy

## Abstract

The progressive and physiological decline in ovarian function depends on the rate of follicular loss by atresia, contributing to the reduction in ovarian reserve. Genetics and environmental factors play important roles in ovarian senescence and in the onset of ovarian dysfunctions such as diminished ovarian reserve. A better understanding of the mechanisms underlying ovarian aging and their regulation by genetic and environmental factors is needed to evaluate ovarian reserve and to predict fertility potential by identification of more accurate and less invasive markers. We report transcriptomic data (i) implicating novel (e.g. EIF2 signalling) and well-known pathways (e.g. TGFβ signalling), and (ii) defining a unique set of non-coding RNA (ncRNA), both associated with ovarian function. The latter includes miRNAs (e.g. *Mir143* and *Mir145*), snoRNAs (e.g. *Snord16a* and *Snora34*), and one lncRNA (*Gas5*), which are differentially expressed in middle-aged ovaries (12 months) *vs* young-aged (3 months) from CD1 mice. Experimental analysis confirms that ovary lifespan varies across genetic backgrounds in mice and, genetics influences the response to environmental perturbations such as diet. Moreover, the identified ncRNAs were verified in a model of reproductive dysfunction promoted by the environmental toxicant ethylenthiourea. We also report the increase of miRNA143 and miRNA145 in follicular fluid of women with diminished ovarian reserve. Their levels inversely correlate with the hormonal profile and with the number of the oocytes recruited upon hormonal stimulation. Overall, we report a transcriptomic signature for ovarian dysfunction in vivo that provides a valuable resource for translational research in human reproductive aging.

## Introduction

The decline in fertility over time results from ovarian aging, which is characterized by quantitative and qualitative alteration of the ovarian reserve^1^. Published reports document that acceleration in the decline of fertility is associated with onset of early menopause, influencing both child bearing and overall women’s health^2^. It has been estimated that 10% of women undergo early menopause before the age of 46, which could correlate with early ovarian aging. Indeed, it has been reported that fertility decreases about 13 years prior to menopause^2^. During this period, ovarian health is declining asymptomatically. Sensitive and specific tests to diagnose this decline are not available^3^.

Genetic and environmental factors affect ovary lifespan by modulating risk of ovarian dysfunctions such as diminished  ovarian reserve (DOR) and premature ovarian failure (POF)^4^. Studies correlating menopausal age with genetic variation suggest a genetic component in determining life of the follicle pool^5^ and reproductive lifespan^6,7^. Notably, mutations in oocyte-derived bone morphogenetic protein 15 (*BMP15*) and in oocyte-derived growth differentiation factor 9 (*GDF9*), both TGF-β family members involved in follicular development, are variably associated with DOR and POF^8^. Inhibin A (*INHA*), a negative modulator of pituitary follicle stimulating hormone (FSH) synthesis, is another candidate gene for POF. Indeed, missense mutations in this gene are associated with POF in humans^9^ and null mutant mice show increased *Fsh* levels and are infertile^10^. Loss of function mutations and single nucleotide polymorphisms in follicle stimulating hormone receptor (*FSHR*) are also linked with ovarian failure by virtue of the gonadotropin resistance that accompanies these mutations^11^.

The importance of genetic components notwithstanding, environmental factors are also emerging as important modulators of ovarian aging/dysfunctions and as major contributors to the increased incidence of female infertility^12^. Furthermore, local living environments are considered a risk factor for women’s fertility, reinforcing the role of environmental exposures in reproductive health^13^. For example, significant levels of persistent endocrine disruptors have been associated with early age at menopause in a large cross-sectional representative sample of US women, leading to DOR and increased risk of infertility^14^.

Despite the dramatic changes that occur as ovaries age, the progressive loss of ovarian function is a silent process^3^. By the time women experience symptoms associated with aging, their fertility has already been severely compromised. Several endocrine markers have been identified and tested in attempts to accurately assess loss of ovarian function over time. Anti-Müllerian hormone (AMH) is proving to be a superior parameter for assessing ovarian dysfunctions^3,15^, especially because AMH levels are independent of the confounding issues associated with phase of the menstrual cycle^16,17^. However, other markers, including inhibins and FSH^18^, are extensively used as indirect reporters of follicle numbers. Furthermore, the antral follicle count (AFC) measured by trans-vaginal ultrasound has been widely used to assess the ovarian reserve, although its accuracy is offset by its invasivity. Despite their applications in the clinic, the accuracy of measurement of these factors is challenging^19,20^. Improvements in the available tests are required for a predictive estimate of the reproductive lifespan and for early diagnosis of individuals affected by ovarian diseases as DOR. This is needed to provide alternative reproductive options before infertility occurs.

Application of “omics” approaches, such as transcriptomic profiling, has proven to be a powerful tool for gaining new insights into mechanisms of disease and identification of possible markers to predict disease prognosis and outcome. Their application to ovarian aging is a rational approach to identify markers with increased predictive power for management of physiological and environmental ovarian dysfunction.

To investigate the utility of transcriptomic markers, we transcriptionally profiled ovaries harvested from young and middle-aged outbred CD1 mice to elucidate mechanisms and identify novel markers of ovarian aging. We report mechanisms underlying physiological decline of ovarian reserve due to aging, and an Ovarian Aging Gene Signature (OAGS), composed of ncRNAs involved in ribosome biogenesis, cell growth and stress response. We report that OAGS is sensitive to genetics, environmental factors (i.e. diet, ethylenthiourea) and gene-environment interactions (GxE), all relevant contributors to ovarian dysfunction and decline of ovarian reserve. Finally, we show that some of the identified ncRNAs (i.e. *MIR143* and *MIR145*) identified in the mouse could be recovered from follicular fluid (FF) of women affected by DOR.

This work increases our understanding of physiological and environmental effects of ovarian dysfunction and reduction in ovarian reserve, which may assist with the development of more accurate diagnostic tools to improve human reproductive biology.

## Results

### OAGS identification and its validation in CD1 mice

**Fig. 1:**
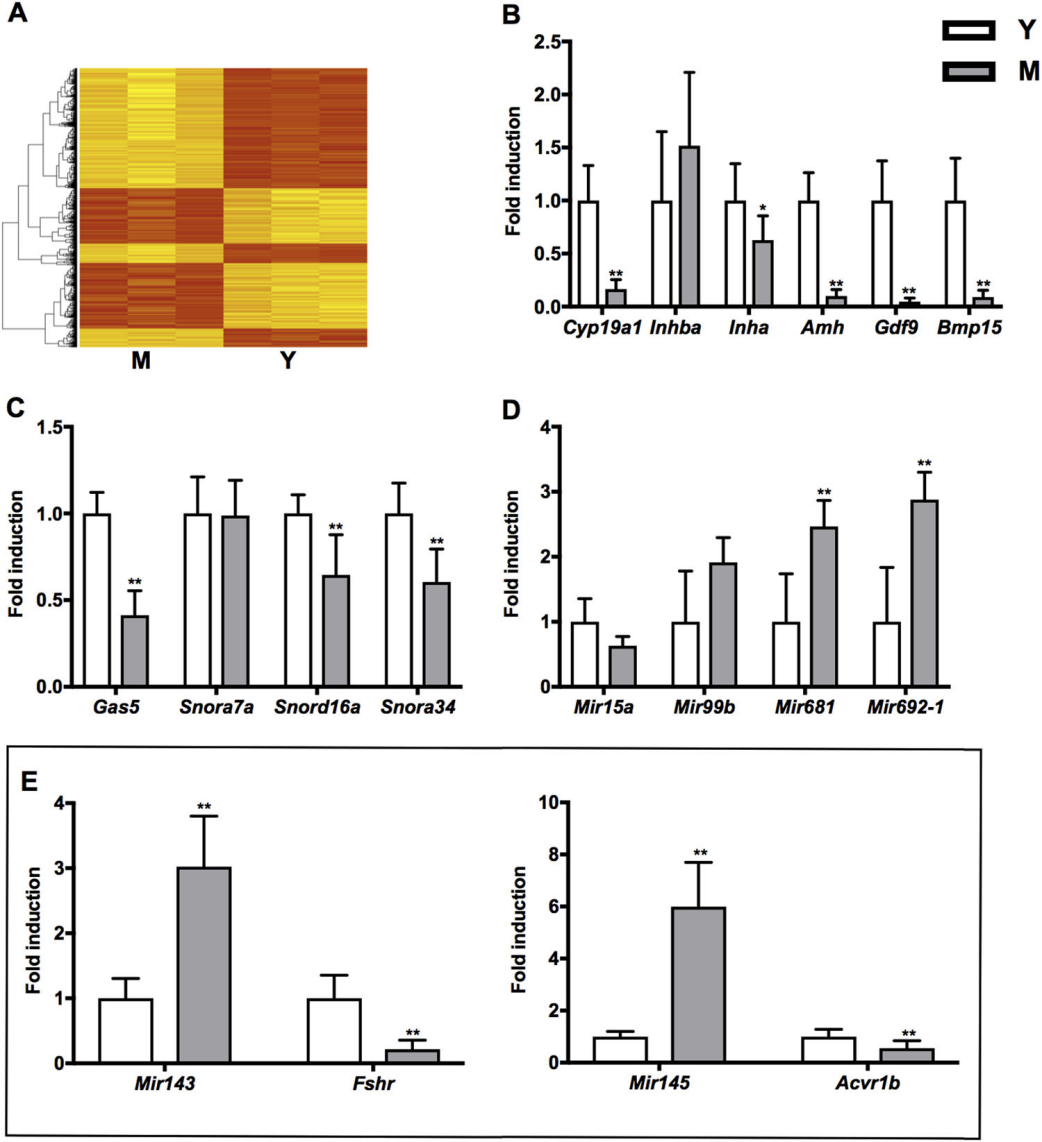
Gene expression analysis identifies an OAGS in female CD1 mice. **A ** Hierarchical clustering of differentially expressed genes in middle-aged (M, *n* = 9) and young (Y, *n* = 9) groups. Microarray was conducted in three samples, each including three animals. **B** qRT-PCR of established markers of ovarian aging. **C, D** qRT-PCR of OAGS consisting of a lncRNA, snoRNAs and miRNAs differentially expressed genes in the two age categories. **E** *Mir143* and *Mir145* and cognate targets *Fshr* and *Acvr1b*, mRNA levels in ovaries of M and Y mice. Data are reported as the ratio between mRNA/miRNA content in M and Y groups normalized to *β-actin*/*Rnu6*. qRT-PCR data are mean ± s.d. with 5–10 animals per group. Significant differences are indicated with *P < 0.05; ** *P* < 0.01

We collected ovaries from 3- and 12-month-old CD1 mice, extracted RNA and performed the gene expression profiling (see Materials and Methods). Substantial differences in gene expression were observed in ovaries from young (Y, 3-month-old) to middle-aged (M, 12-month-old) mice (Fig. 1A). We identified 974 differentially expressed genes (DEGs) between the two samples. Confidence in the differential gene set was enhanced by the fact that among genes with reduced expression were transcripts previously associated with ovarian aging: anti-Müllerian hormone (*Amh*, logFC −1.01, *P* 4.58E-02), follicle stimulating hormone receptor (*Fshr*, logFC −1.49, *P* 4.66E-02) and bone morphogenetic protein15 (*Bmp15*, logFC −1.40, *P* 2.62E-02). We verified by qRT-PCR the differential expression of these genes and of other established markers of physiological ovarian aging: aromatase, *Cyp19a1*; activin-βA, *Inhba*; inhibin-α, *Inha*; growth differentiation factor 9, *Gdf9* (Fig. 1B and Table S2, Supplementary information). As expected, most of the transcripts were reduced in the M *vs* Y group with varying levels of statistical significance (Table S2, Supplementary information). Specifically, *Cyp19a1, Inha*, *Amh*, *Gdf9* and *Bmp15* transcript levels were diminished whereas *Inhba* transcript level showed a trend toward upregulation (Fig. 1B). Functional bioinformatic analyses using Ingenuity Pathway Analysis (IPA) enriched for pathways listed in Table 1. Among them, some have a well-established association with ovarian aging (e.g. TGF-β signalling), while others have been recently associated with it (e.g. mitochondrial dysfunction)^21^. Some others have not been reported yet with ovarian aging such as stress response signalling. Notably, genes identified within the stress response pathway are known to modulate the translation process. These include transcripts encoding ribosomal proteins (RPs) and small nucleolar RNAs (snoRNAs), suggesting impairment of ribosome assembly. Components of the translational machinery (e.g. *Eif3a*, *Eif3m* and *Eif4g2*), the ribosomal proteins (e.g. *Rps3* and *Rps24*) and small nucleolar RNAs (e.g. *Snora7a*, *Snord16a* and *Snora34*) were significantly down regulated in M- ovaries by microarray and by qRT-PCR (Table S3, Supplementary information). snoRNAs and long non-coding RNA growth arrest-specific 5 (*Gas5*) transcripts were significantly decreased in M- ovaries with the only exception being *Snora7a* (Fig. 1C). *Mir143*, *Mir145, Mir681* and *Mir692-1* were significantly induced in M *vs* Y ovaries (Fig. 1d). *Mir15a* failed to pass the qRT-PCR validation criterion while *Mir99b* induction was not significant (Fig. 1D). miRNAs induction in M- ovaries was pertinent since they exert a crucial role in ovarian activity by regulating different steps of follicle recruitment and maturation^22^. *Mir143* and *Mir145* regulate follicle maturation by targeting the *Fshr* and Activin A receptor type 1b (*Acvr1b*), respectively^23,24^. Quantitative PCR confirmed a relevant down regulation of both targets in M- ovaries (Fig. 1E).

**Table 1:** Functional annotation analysis of deregulated transcripts obtained comparing M and Y mice ovaries. Transcripts were classified according to canonical pathways using IPA bioinformatics tool

Ingenuity canonical pathways	−log (*P*-value)	Downregulated	Upregulated
EIF2 signalling	1.79E01	37/181 (20%)	3/181 (2%)
Regulation of eIF4 and p70S6K signalling	1.16E01	25/149 (17%)	4/149 (3%)
Mitochondrial dysfunction	1.09E01	22/159 (14%)	7/159 (4%)
Oxidative phosphorylation	1.01E01	20/99 (20%)	2/99 (2%)
Protein ubiquitination pathway	9.28E00	30/245 (12%)	4/245 (2%)
mTOR signalling	7.72E00	22/190 (12%)	5/190 (3%)
Mitotic roles of polo-like kinase	3.57E00	10/64 (16%)	0/64 (0%)
Unfolded protein response	2.86E00	6/53 (11%)	2/53 (4%)
TCA Cycle II (Eukaryotic)	2.77E00	5/22 (23%)	0/22 (0%)
Pyrimidine ribonucleotides de novo biosynthesis	2.69E00	3/33 (9%)	3/33 (9%)

**Fig. 2:**
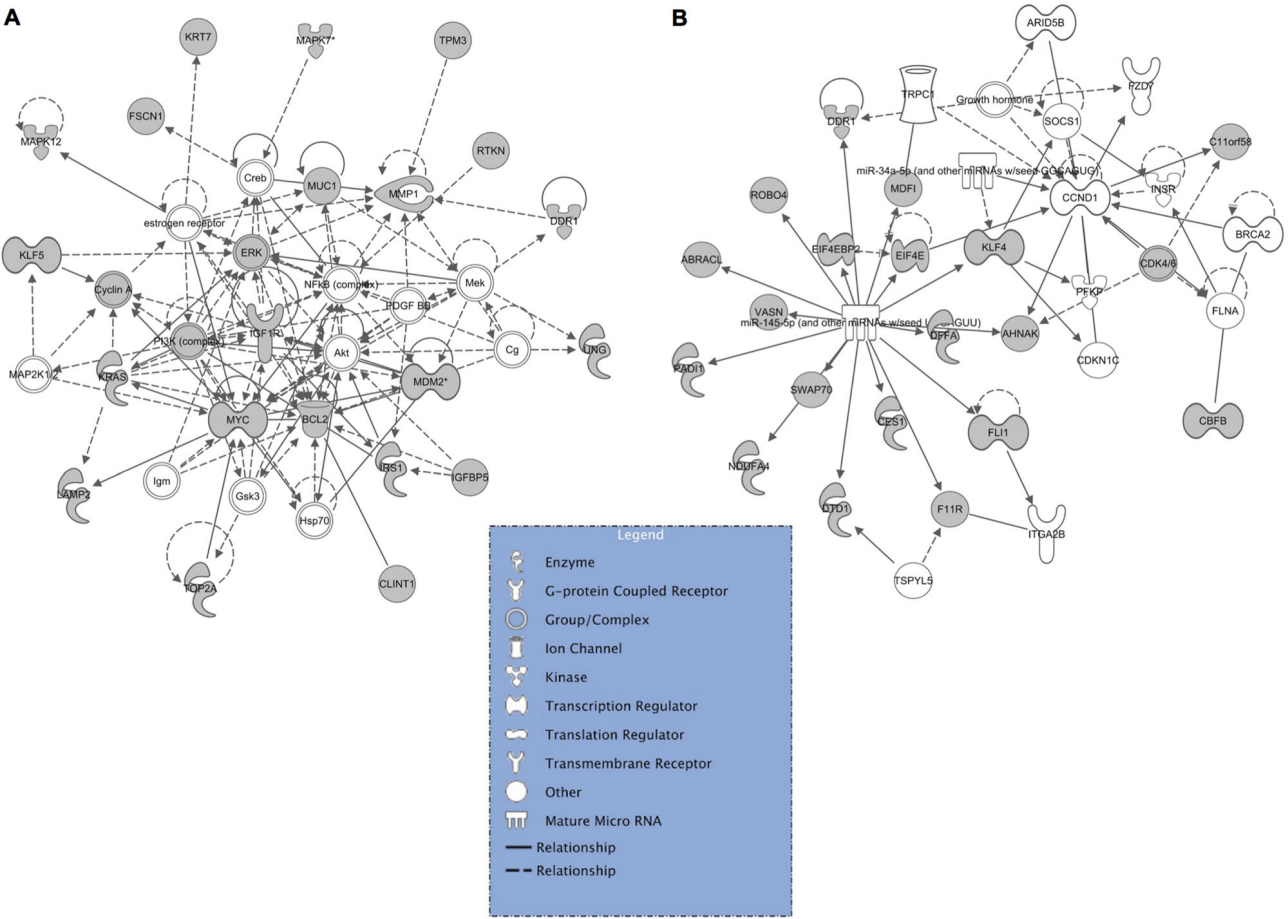
Networks of putative target genes regulated by differentially expressed miRNAs. Validated target genes (grey nodes) mapped to pathway annotations derived from literature and gene ontology using Ingenuity Pathway Analysis (IPA, http://www.ingenuity.com) **A** *Mir143* regulatory network **B** and *Mir145* regulatory network. The solid lines connecting molecules represent a direct relation and dotted lines an indirect relation. IPA constructs networks that optimize for both interconnectivity and number of Focus Genes (the grey nodes) under the constraint of a maximal network size. White nodes are added by the algorithm to build a highly connected molecular network between Focus Genes

Further bioinformatics analyses using IPA were performed to identify networks of putative targets of the identified miRNAs. Since IPA software uses data already published, networks were obtained only for *Mir143* and *Mir145* (Fig. 2A, B). Interestingly, some DEGs identified in our data set were retrieved among the putative targets of *Mir143* and *Mir145*, such as *Ndfua4* (logFC −2.18, *P* 5.12E-03) and *Top2a* (logFC −1.43, *P* 1.00E-02), respectively.

We assembled ncRNAs into an Ovarian Aging Gene Signature (OAGS) and reported its components in bold in Table S3, Supplementary information. It comprises (i) miRNAs (*Mir143*, *Mir145*, *Mir505*, *Mir681* and *Mir692-1*), since these are particularly amenable to detection in human fluids (e.g. follicular fluid) and hold greatest potential as biomarkers; (ii) snoRNAs (*Snord16a* and *Snora34*), involved in stress response; (iii) *Gas5*, a lncRNA associated with poor prognosis in human ovarian cancer^25^.

### OAGS in genetically diverse mice

Genetic factors are recognized as major contributors of early reduction in ovarian reserve. To investigate their role, we performed OAGS profiling in C57BL/6J and FVB/NJ mouse strains that show variability in oocyte development and in oestrogen sensitivity^26^. Ovaries were collected from 8-month-old females of both strains, used in a study aimed to assess the GxE interaction in metabolic disease development^27^. This age was expected to correspond to the age at which ovarian reserve begin to decline in women^28^. This was confirmed by the evaluation of the genetic markers of ovarian function (Table S2, Supplementary information) assessed by qRT-PCR (Table 2). Depending on their different genetic backgrounds, FVB/NJ mice had decreased expression of all established markers of ovarian aging (*Amh*, *Gdf9*, *Bmp15* and *Inha*) compared to C57BL/6J mice of the same age (Table 2). Expression of the ncRNAs included in the OAGS was also verified in both strains. *Gas5*, *Snord16* and *Snora34* transcripts showed a general downregulation in ovaries from FVB/NJ mice compared to ovaries from C57BL/6J mice (Table 2). We further examined whether miRNAs *Mir681*, *Mir692-1*, *Mir143* and *Mir145* were induced in these strains. *Mir681* and *Mir692-1* were significantly induced in FVB/NJ relative to C57BL/6J (Table 2). *Mir143* expression was also significantly induced in FVB/NJ. This effect translated to a significant downregulation of its target *Fshr* (Table 2). Surprisingly, *Mir145* was generally upregulated in FVB/NJ compared to C57BL/6J but the expected effect on downregulated expression of *Acvr1b* expression was not observed (Table 2).

**Table 2:** Established markers of ovarian aging and OAGS expression in genetically diverse mice

GeneSymbol	GeneDescription	Relative expression in C57BL/6J^a^ (mean ± s.d.)	Relative expression in FVB/NJ^a,b^ (mean ± s.d.)
*Cyp19a1*	Cytochrome P450, family 19, subfamily a, polypeptide 1	0.06 ± 0.012	0.033 ± 0.014
*Inhba*	Inhibin beta-A	0.023 ± 0.074	0.103 ± 0.054
*Inha*	Inhibin alpha	0.360 ± 0.094	0.195 ± 0.044**
*Amh*	Anti-Mullerian hormone	0.163 ± 0.041	0.091 ± 0.029**
*Gdf9*	Growth differentiation factor 9	0.028 ± 0.006	0.018 ± 0.004**
*Bmp15*	Bone morphogenetic protein 15	0.015 ± 0.003	0.011 ± 0.003**
***Gas5***	Growth arrest specific 5	1.784 ± 0.310	1.589 ± 0.181
***Snord16a***	Small nucleolar RNA, C/D box 16A	0.113 ± 0.047	0.114 ± 0.022
***Snora34***	Small nucleolar RNA, H/ACA box 34	1.218 ± 0.224	1.185 ± 0.296
***Mir143***	microRNA 143	0.006 ± 0.002	0.117 ± 0.026**
***Mir145***	microRNA 145	335.0 ± 189.0	2868.0 ± 562.0
***Mir681***	microRNA 681	0.181 ± 0.113	1.849 ± 0.402**
***Mir692-1***	microRNA 692-1	0.059 ± 0.029	1.877 ± 0.540**
*Fshr*	Follicle stimulating hormone receptor	0.023 ± 0.004	0.009 ± 0.002**
*Acvr1b*	Activin A receptor, type 1B	0.005 ± 0.001	0.007 ± 0.002

^a^2^−∆∆Ct^

^b^*P* < 0.05; **P < 0.01 relative expression in FVB/NJ/C57BL/6J *P < 0.05; **P < 0.01 relative expression in FVB/NJ/C57BL/6J

Overall, the data suggest genetics as factor influencing the variability between the chronological and real biological ovarian age.

### OAGS in mice exposed to environmental factors promoting ovarian dysfunction

Environmental factors, such as lifestyle and diets might cooperate with genetic factors in regulating ovarian function; hence we examined the effects of different diets on ovarian aging as a function of genetic background. Cohorts of 6 weeks old C57BL/6J and FVB/NJ mice were provided *ad libitum* access to rodent chows that model those of Mediterranean, American and ketogenic diets up to 8 months of age (Fig. S2, Supplementary information)^27^.

**Fig. 3:**
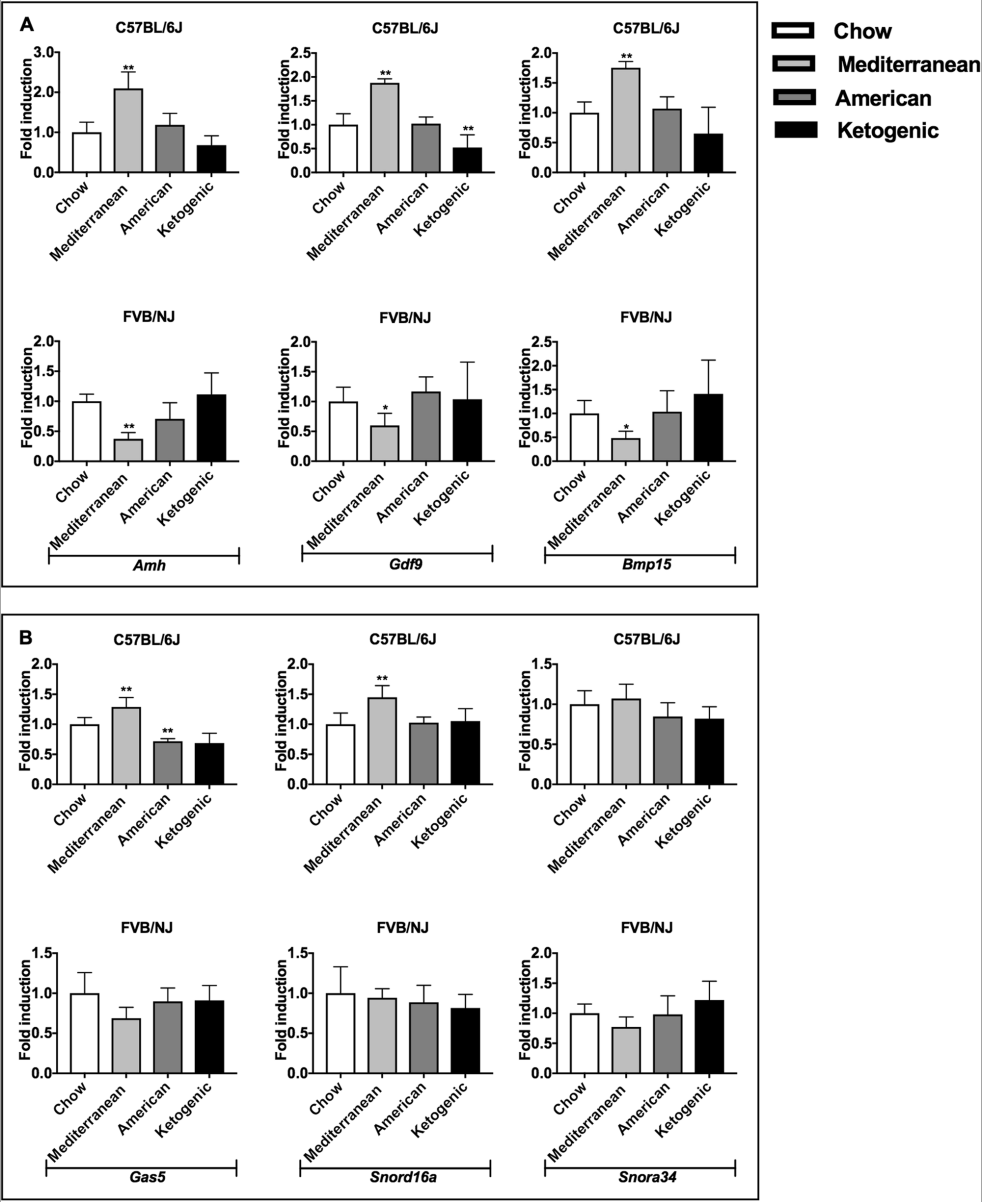
OAGS changes in C57BL/6J and FVB/NJ mice fed different diets. **A** Dietary effects on ovarian reserve were determined by qRT-PCR of established ovarian marker *Amh, Gdf9, Bmp15* in both strains fed with different diets from weaning to necropsy. **B** OAGS downregulated genes were verified by qRT-PCR in both strains. Data are reported as the ratio between mRNA content exposed and control groups normalized to *β-actin*. Data are mean ± s.d. with five animals per group. Significant differences are indicated with *P < 0.05; ** *P* < 0.01

Markers of ovarian function were tested in both strains fed different diets (Fig. S3, Supplementary information). Notably, *Amh, Gdf9, Bmp15* expression was found generally upregulated in C57BL/6J mice when fed Mediterranean and American diets compared to the control group fed a rodent chow diet. By contrast, the same transcript levels in ovaries from FVB/NJ mice were generally reduced when fed Mediterranean or American diets compared to rodent chow and showed a trend toward upregulation when fed the ketogenic diet (Fig. 3A).

**Fig. 4:**
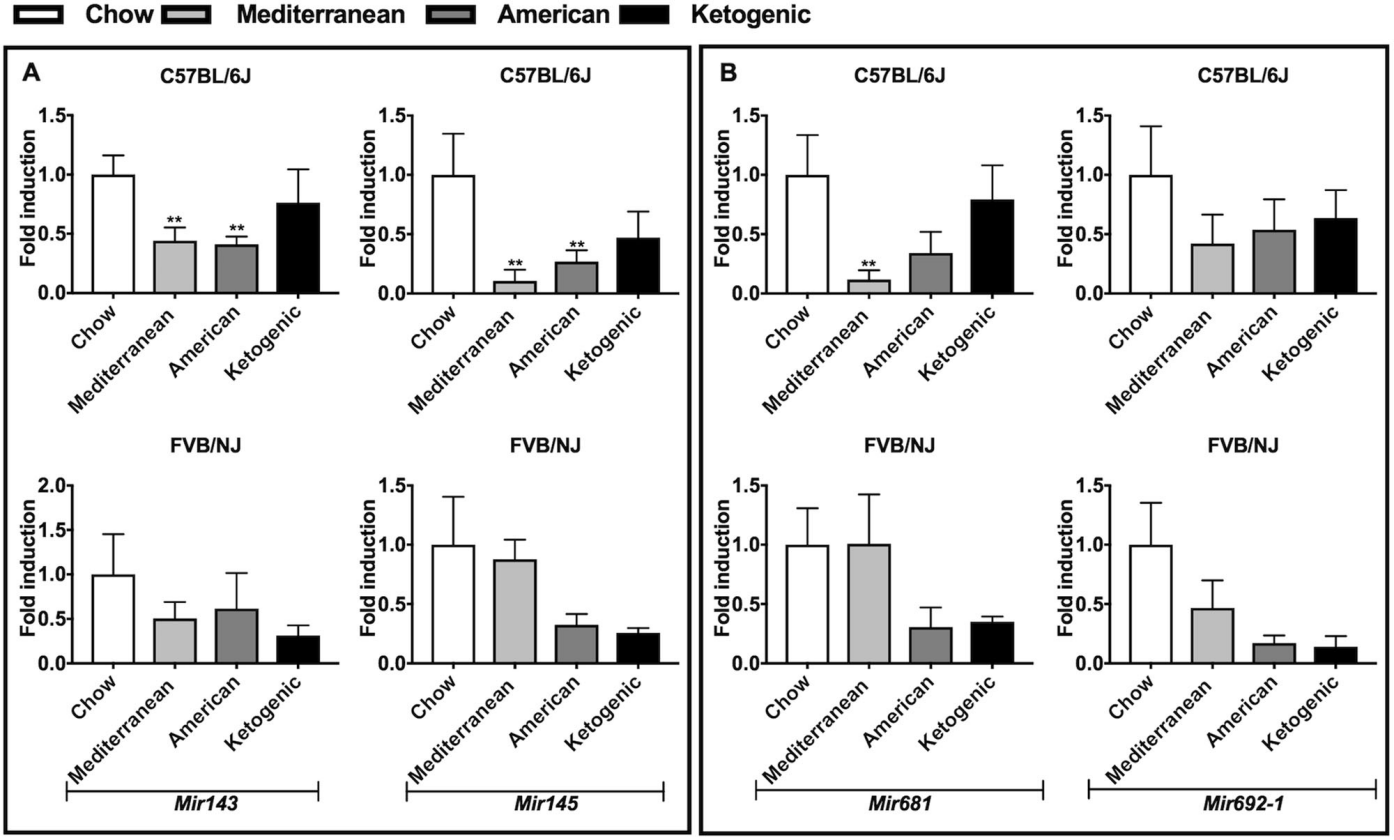
miRNAs changes in C57BL/6J and FVB/NJ mice fed different diets. Dietary effects on OAGS miRNAs were determined by qRT-PCR in C57BL/6J and FVB/NJ mice fed with different diet from weaning to necropsy. **A** *Mir143* and *Mir145* expression levels were verified in both strains. **B** *Mir681* and *Mir692-1* expression levels were verified in both strains. Data are reported as the ratio between miRNA content exposed and control groups normalized to *Rnu6*. Data are mean ± s.d. with five animals per group. Significant differences are indicated with *P < 0.05; ** *P* < 0.01

OAGS was also evaluated in females of both strains fed different diets. We confirmed a general increased expression of the OAGS signature in the ovaries of C57BL/6J females fed the Mediterranean diet, although significance was reached only for *Gas5* and *Snord16a*. Furthermore, *Gas5* transcript was significantly downregulated in ovaries from C57BL/6J females when fed the American diet, suggesting its detrimental effect on ovarian function. By contrast, OAGS showed no relevant changes in ovaries from FVB/NJ mice, although the observed trends toward downregulation of *Gas5*, *Snord16a* and *Snora34* could indicate a detrimental effect of Mediterranean diet (Fig. 3B). Surprisingly, *Mir143* and *Mir145* were downregulated in the ovaries of C57BL/6J when fed any diet compared to rodent chow (Fig. 4A), although statistically significant only for C57BL/6J females fed the Mediterranean or American diets. The same trend was retrieved in ovaries from FVB/NJ. Furthermore, *Mir681* and *Mir692-1* were generally downregulated as a function of diet in both strains, with the most significant differences detected for *Mir681* in ovaries of C57BL/6J mice fed with Mediterranean diet. By contrast, *Mir681* showed no difference in FVB/NJ mice on the Mediterranean diet (Fig. 4B). Interestingly, miRNAs showed a trend toward downregulation in ovaries of FVB/NJ mice when fed the ketogenic diet, although not significant.

These data suggest that genetic background influences ovarian function and, in concert with exposure to environmental stressors such as diet, these factors can either positively or negatively affect ovary lifespan. Taken together, exposure to American diet was frequently mixed in its effects, while the Mediterranean and ketogenic diets seem to differentially impact ovarian function in C57BL/6J and FVB/NJ, respectively.

**Fig. 5:**
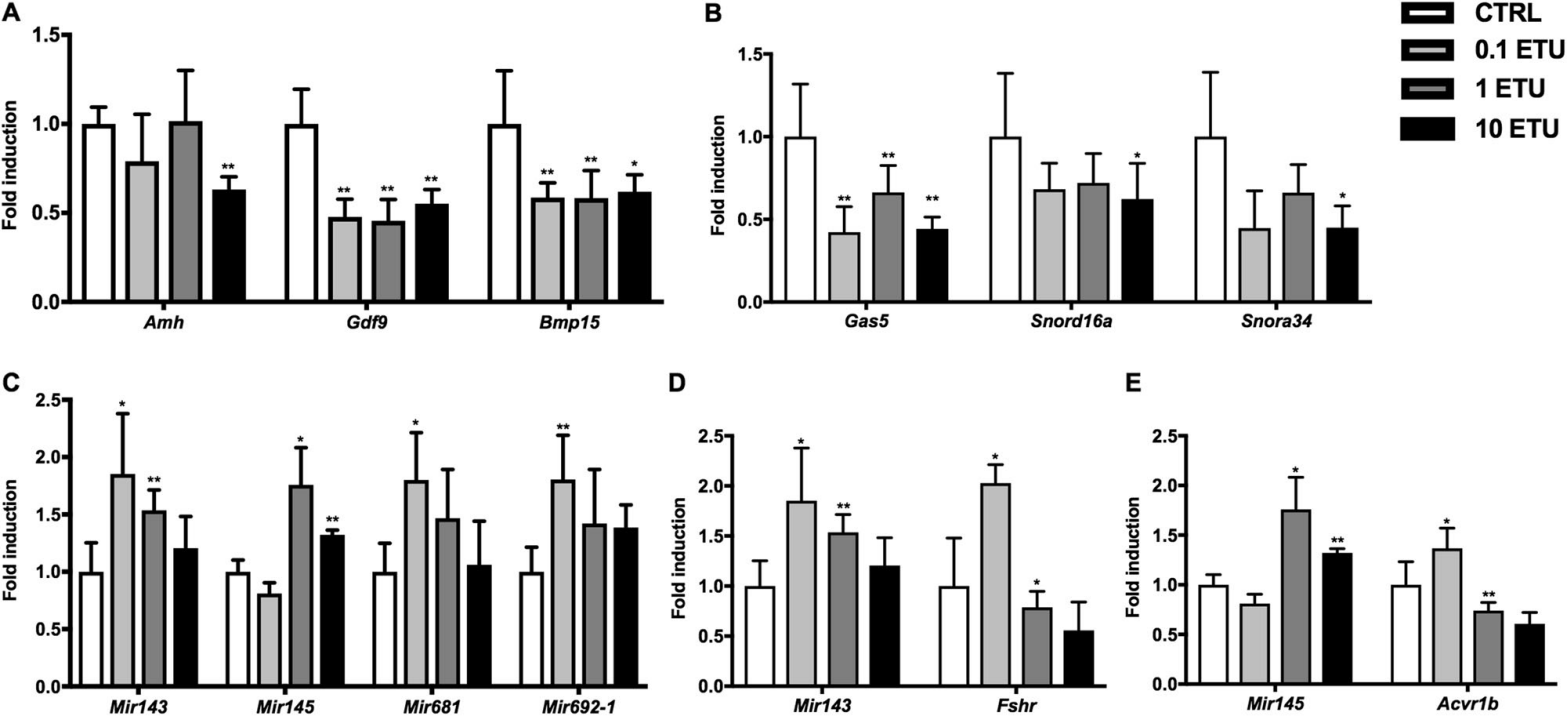
OAGS changes in CD1 mice exposed to ETU since the conception. **A **ETU effects on ovarian reserve were determined by qRT-PCR of established ovarian marker *Amh, Gdf9, Bmp15*, in mice exposed from the GD0 to PND180 (Fig S1, Supplementary information). **B **ETU-induced ovarian dysfunction was assessed by testing the OAGS across all doses of ETU by qRT-PCR. **C** miRNAs included into the OAGS were tested by qRT-PCR in all groups. **D, E ***Mir143/Mir145* and cognate targets expression levels were determined by qRT-PCR. Data are reported as the ratio between mRNA/miRNA content in exposed and control groups normalized to *β-actin/Rnu6* and, are mean ± s.d. with five animals per group. Significant differences are indicated with *P < 0.05; ** *P* < 0.01

Environmental toxicants can affect ovarian health. Developmental exposure to low-dose ethylenthiourea (ETU) is associated with prolonged oestrous cycle in female rats, suggesting an early reproductive senescence^29^ that compromises the programming of the female reproductive system^30^. Mancozeb, an ethylene bis-dithiocarbamate whose main metabolite is ETU, exerts dose-dependent toxic effects on granulosa cells (GCs) and oocytes in vitro^31^. These alterations may represent a major cause of infertility associated with exposure to pesticides^32,33^. Therefore, the OAGS was evaluated in ovaries from CD1 mice exposed to environmentally relevant doses of ETU (0.1, 1, and 10 mg/kg/day, by drinking water) from conception to 6 months of age (Fig. S4, Supplementary information). *Amh*, *Gdf9* and *Bmp15* transcript levels were evaluated in mice exposed to ETU to determine its toxic effects on follicle maturation (Fig. 5A). *Amh* was significantly decreased at the highest dose, whereas *Gdf9* and *Bmp15* were significantly downregulated at all doses (Fig. 5A). The ncRNAs of the OAGS showed a trend toward downregulation in ovaries of mice exposed to all doses of ETU at 6 months of age, compared to the control group (Fig. 5B). Specifically, *Snord16a* and *Snora34* were significantly down regulated only at the highest dose while the lncRNA *Gas5* was significantly decreased in all exposed groups (Fig. 5B). We confirmed the induction of *Mir143*, *Mir145*, *Mir681* and *Mir692-1* in ETU exposed ovaries (Fig. 5C). Remarkably, *Mir143*, *Mir681* and *Mir692-1* expression levels were increased in all exposed groups, although statistically significant differences were observed at the lowest two doses of ETU for *Mir143* and only at the lowest dose for *Mir681* and *Mir692-1* (Fig. 5C). *Mir145* expression, however, was only induced in mice exposed to the two highest doses of ETU (Fig. 5C). *Fshr* transcript was decreased in ovaries from females exposed to the two highest doses of ETU but induced at the lowest dose of ETU (Fig. 5D). Although the *Acvr1b* expression levels were increased in the ovaries of mice exposed to the lowest dose of ETU, they were significantly diminished at the two highest doses, as expected (Fig. 5E).

Overall, the OAGS seems to be more sensitive than *Amh* in this exposure model since its impairment is evident at low dose as well as high-dose ETU exposures.

### *MIR143* and *MIR145* levels are increased in follicular fluid from women with diminished ovarian reserve

**Fig. 6:**
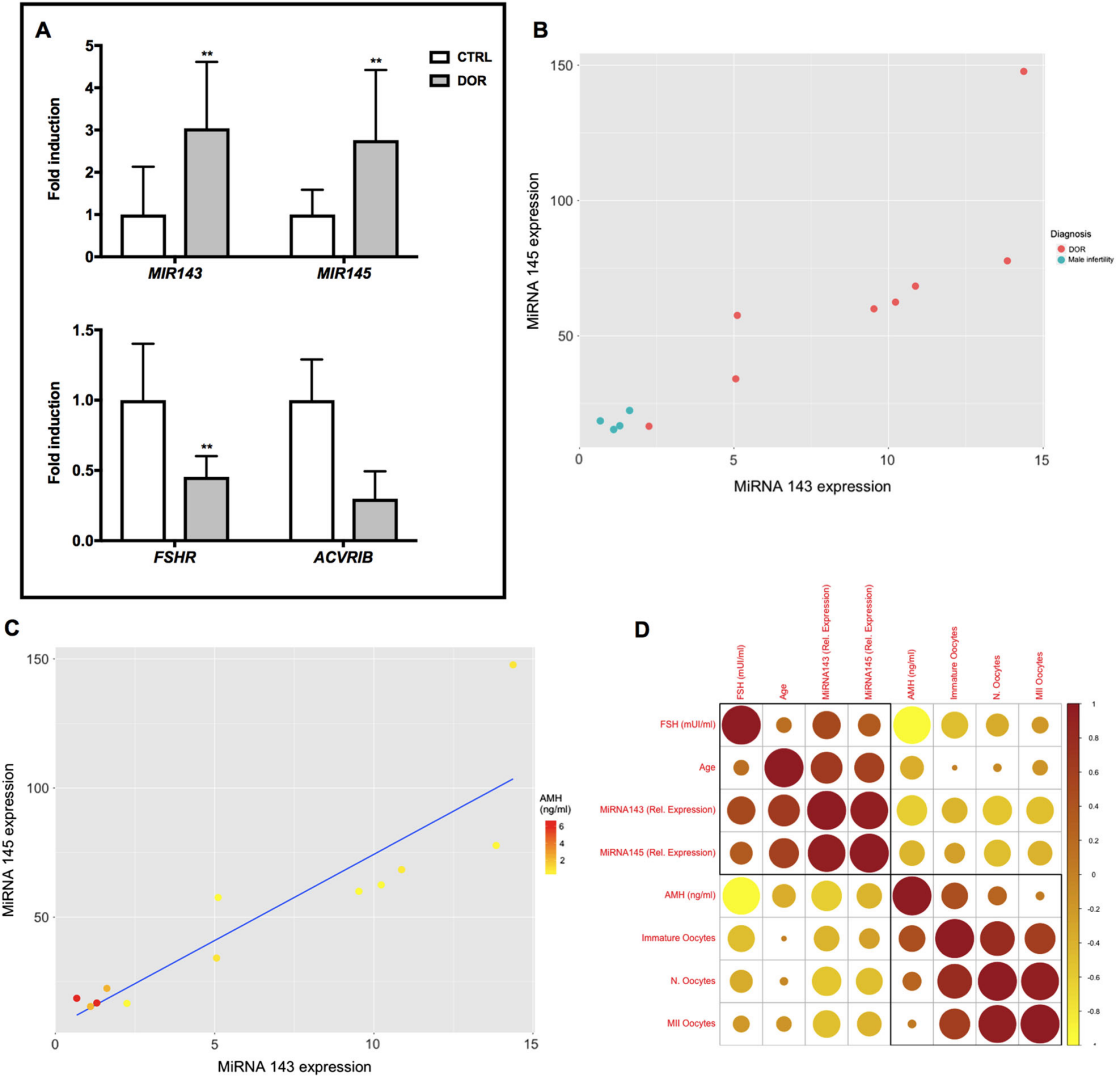
miRNAs aging-related expression in FF of women with DOR. **A ***MIR143* and *MIR145* levels were tested by qRT-PCR in FF from DOR-affected and healthy women. *FSHR* and *ACVRIB*, respectively, were evaluated by qRT-PCR in GCs isolated from FF of women with DOR and healthy women. Data are reported as the ratio between mRNA/miRNA content exposed and control groups normalized to *β-ACTIN*/*RNU6*. Data are mean ± s.d. with 5 pz per control group and 7 pz DOR-affected. Significant differences are indicated with *P < 0.05; ** *P* < 0.01. **B **Scatterplots of relative expression show the correlation between *MIR143* and *MIR145* and DOR diagnosis and **C **AMH serum levels. **D** Spearman’s rank correlation illustrated as corrplot. Brown circles indicate positive correlation. Yellow circles illustrate negative correlation. The colours are linked to values between −1 (yellow) and 1 (brown) using the scale on the side. Size of the circles corresponds to absolute value of the correlation coefficients

Diminished ovarian reserve is increasing in incidence, and has a complex aetiology that includes both genetic and environmental factors. We only evaluated *MIR143* and *MIR145* in follicular fluid (FF) of women affected by DOR as proof-of-principle for their use as biomarkers. *Mir681* and *Mir692-1* were excluded because they do not have human orthologs. *MIR143* and *MIR145* expression was monitored in FF from an experimental cohort of women pursuing to assisted reproductive technology (ART) with signs of DOR, whereas women pursuing to ART as a consequence of male infertility represented the control group. *MIR143* and *MIR145* both showed a significant increase in FF from women with DOR compared to healthy women (Fig. 6A). We also verified the expression of *Mir143* and *Mir145* targets in GCs isolated from the FF, representing the GC cells of the cumulus oophorus. Both target sets were also downregulated although statistical significance was reached only for *FSHR* transcript (Fig. 6A).

We further investigated the association of *MIR143* and *MIR145* with DOR diagnosis, using Spearman’s rank correlation (Fig. 6B). Increase of both miRNAs discriminates DOR from healthy women. Moreover, increased expression of mature *MIR143* and *MIR145* is associated with serum-reduced levels of AMH (Fig. 6C) and elevated levels of FSH (Fig. S1, Supplementary information). We then determined the association of both miRNAs with other DOR-associated parameters (i.e. number of oocytes collected and number of oocytes at MII stage). The correlation plot (Fig. 6D) suggests that elevated expression of both miRNAs negatively correlate with total number of collected oocytes and with the mature ones. These data reveal a clear association between *MIR143* and *MIR145* and DOR in humans.

## Discussion

Ovarian aging occurs progressively and has a complex aetiology that includes genetic, epigenetic and environmental factors^34,35^. Because of this complexity and of its progressive and asymptomatic onset, clinical diagnosis remains a challenge. An understanding of the molecular mechanisms that operate during the ovarian aging are needed to support development of diagnostic tests that can accurately assess the ovarian reserve and predict its decline.

Using transcriptome profiling to compare young- and middle-aged ovaries in mice, we identified a distinct ovarian aging gene signature (OAGS) that is not only consistent with what is currently known about the ovarian aging process, but it also identifies novel candidate biomarkers of ovary lifespan (e.g. circulating ncRNAs). Validation experiments show that the OAGS is sensitive to both genetic and, at least, two independent environmental variables (diets and ETU) suggesting that the signature has the potential to greatly improve prediction and diagnosis of physiological and environmental decline of ovarian reserve. Analyses of follicular fluid from humans propose the OAGS as a promising translational test for developing an effective and predictive biomarker platform to monitor ovarian health and age in humans.

The OAGS was identified by bioinformatic analysis of transcriptomic data revealing differentially expressed genes in pathways, including EIF2 signalling, mTOR signalling, mitochondrial stress pathways and TGF-beta signalling^21,36–38^. Notably, the bioinformatics analyses of our data set pointed out the deregulation of the protein translation machinery^39^. These data forecast that ovaries of middle-aged females are compromised for bulk protein synthesis. Such a phenotype is consistent with elevated cellular stress^40^: cells devote their diminished translational capacities to the selective translation of proteins vital for stress management and recovery^41^. The fact that stressful conditions, such as increased oxidative stress, occur in ovarian aging is consistent with the reported profiling data, corroborating a major role of stress signalling in promoting physiological depletion of ovarian reserve^42^. This is in agreement with other transcriptomic profiling studies in animal models linking endoplasmic reticulum stress (eIF2 signalling), activities of the cellular translational machinery and mitochondria to ovarian aging and oocyte reprogramming^21,43^. Interestingly, analyses of transcripts targeted by *Mir143* and *Mir145* (Fig. 2) provide evidence for their involvement in electron transport chain (i.e. *Ndfua4)*^44^ and in oocytes reprogramming (i.e. *Top2a*)^35^.

Although it remains unclear how these pathways modulate physiological and environmental induced ovarian dysfunction, these data have the potential for establishing a novel mechanistic-based diagnostic platform for monitoring ovarian health and aging by testing ncRNAs, since they are easily detectable in human fluids. Recent studies have shown that miRNAs regulate follicle growth and maturation^45^ and *Mir143* and *Mir145* downregulate, respectively, *Fshr* and *Acvr1b* transcripts^24,42^. Noteworthy, the reduced *Fshr* expression is associated with oxidative stress in GCs^45^, a result consistent with our data implicating stress pathways activation as promoters of reduced ovary lifespan. Although miRNA143 and miRNA145 have been detected in FF^23,24^, our data represent the first evidence for their association with ovarian aging and DOR. Although a larger sample size would improve the significance, the data nonetheless suggest that the expression of both miRNAs negatively correlate with the number of mature oocytes. In agreement with this observation, *Top2a,* a putative target of *Mir145* reported as a major player in the reprogramming properties of oocytes^46^ was among the inhibited transcripts in our data set.

The data are corroborated by further analyses assessing cooperation of genetic background and environmental factors in determining diminished ovarian reserve. In agreement with previous reports, the OAGS confirmed that diet plays a role in modulating ovarian reserve, but as a function of genetic background, which is the major contributor. Our data suggest that OAGS change is more sensitive than *Amh* in mouse ovaries of females exposed to different doses of ETU.

Overall, the reported data display that comprehensive characterization of ncRNAs, miRNAs, as well as their regulators and related gene networks affected by female age represent a valuable resource to investigate the biology of ovarian dysfunction due to gene-environment interactions, both key determinants of ovary lifespan. We identified biomarkers sensitive to GxE interactions, which are applicable to humans. Although the use of rodents for ovarian dysfunction studies is controversial, our data strongly suggest that mice are a valuable model if the data are properly controlled and the experiments appropriately designed^47^.

## Materials and methods

### Ethics statement

All animal experiments were performed in accordance and approval by Health Ministry (ID number 1-2010) and Texas A&M University Institution of Animal Care and Use Committee (ID number 0182-2013, 0339-2016). Use of patient and clinical material was approved by the Ethics Committee of the Azienda Ospedaliera San Giuseppe Moscati (Avellino, Italy; reference number CE 6-2009, approval granted 5 June 2009). Informed consent was obtained from all participants.

### Animal treatments

The microarray experiment was performed in female CD1 mice aged 3 (Y) and 12 months (M) of age. Mice were housed in groups of 5 at 22 ± 2 °C under 12 h light/12 h dark cycle. Animals received water and standard rodent pelleted chow (4RF21 form Mucedola) *ad libitum*.

Ethylenthiourea (ETU) exposure was performed in female CD1 mice 7 days before the mating. ETU was administered through drinking water at 0.1, 1 and 10 mg/kg/day until weaning. The offspring were exposed through the mothers from gestational day 0 (GD0) till postnatal day 21 (PND21) and then directly up to 6 months of age (Fig. S4, Supplementary information).

Exposure to different diets was performed in female C57BL/6J and FVB/NJ mice, obtained from The Jackson Laboratory (Bar Harbor, ME) at four weeks of age. Mice were allowed 2 weeks for acclimation before being assigned to a diet group. Mice were housed 5 females for strain and diet per cage and kept in a facility in Texas A&M University. Mice were euthanized at 8 months of age (Fig. S2, Supplementary information). Powdered diets were designed with the assistance of Research Diets, Inc. (New Brunswick, NJ). Traditional Mediterranean (D12052702) diet was based on the Food and Agriculture Organization’s Food Balance Sheets from Greece and Japan in 1961. The American diet (D12052705) was based on USDA’s 2008 Dietary Assessment of Major Food Trends. The Ketogenic diet (D12052706) was adapted from a previous study, with menhaden oil added to provide additional omega-3 fatty acids^48^. Detailed composition of the diets can be found in Barrington et al.^27^. Standard mouse chow (D12052701, Research Diets, Inc.) was used as a control diet. Diets were designed to recapitulate human diets as closely as possible, matching macronutrient ratio, fiber  content, types of ingredients and fatty acid ratios to the human diets^27^.

Mice in all studies were euthanized by carbone dioxide inhalation. Ovaries were collected and used for the experiments as described below.

### Participants

After obtaining informed consent, FF samples were collected from twelve patients scheduled for IVF (age range 32–44 years old). DOR was diagnosed according to the Bologna criteria^49^. Inclusion criteria were advanced maternal age (≥40), AFC ranging from 0 to 7, AMH ranging from 0 to 1.1 ng/ml and FSH ≥ 12 mUI/ml. For comparison, patients with severe male factor infertility were set as a control group (*n* = 5). We excluded patients suffering from other related disorders, such as polycystic ovary syndrome, Turner syndrome, thyrotoxicosis, hyperprolactinemia or recurrent spontaneous abortion, as well as patients who had undergone ovarian surgery or chemotherapy.

### Collection of GCs and FF

Samples of follicular fluid (FF) from large follicles (diameter > 16 mm) were collected and separately examined to detect immature and mature oocytes (see Supplementary table S4). Fluids collected from a single patient were pooled. GCs from each patient were isolated by centrifugation at 1200 rpm for 5 min at RT, after removal of red blood cells. Samples were frozen at −80 °C until RNA isolation.

### RNA isolation

RNA was isolated from ovaries using different protocols depending on the study. *CD1 mice ovary RNA isolation*. RNA was isolated from CD1 mouse ovaries using Trizol reagent (Invitrogen) according to the manufacturer’s protocol. *Human samples RNA isolation*. The same procedure, described for CD1 mice ovary RNA isolation was applied to prepare RNA from human GCs obtained by centrifugation of FF. *C57BL/6J and FVB/NJ mice ovary RNA isolation*. RNA was isolated from C57BL/6J and FVB/NJ mouse ovaries using Maxwell® 16 LEV simplyRNA Purification for tissues (Promega) according to the manufacturer’s protocol.

### cDNA synthesis and qRT-PCR for mRNA detection

qRT-PCR were performed using different protocols depending on the study. *CD1 mice ovary cDNA synthesis and qRT-PCR*. Reverse transcription, primers design and qPCR were performed using QuantiTect Reverse Transcription Kit (Qiagen), NCBI Primer Blast and Power SYBR Green Master Mix (Applied Biosystems with Applied Biosystem 7900 Real-Time PCR System), respectively. qRT-PCR analysis was performed in triplicate. Data were normalized to β-actin (*Actb*) transcript levels. The 2^−∆∆Ct^ method was used to calculate relative expression changes with a control group as a reference point^50^. *Human samples cDNA synthesis and qRT-PCR*. The same procedure, described for CD1 mice ovary cDNA synthesis and qRT-PCR was applied to human GCs. Data were normalized to β-actin (*ACTB*) transcript levels. *C57BL/6J and FVB/NJ mice ovary cDNA synthesis and qRT-PCR*. Reverse transcription, primers design and qPCR were performed using the Transcriptor First Strand cDNA Synthesis Kit (Roche Diagnostics), NCBI Primer Blast and LightCycler® 480 SYBR Green I Master (Roche Diagnostics with LightCycler® 96 System), respectively. Data were normalized as described above. qPCR primers are listed in Table S1, Supplementary information.

### miRNA isolation

miRNAs were isolated from ovaries using different protocols depending on the study. *CD1 mice ovary miRNA isolation*. miRNAs were isolated from CD1 mouse ovaries using Trizol reagent (Invitrogen) and, then, enriched using the miRNeasy Mini Kit (Qiagen) according to the manufacturer’s protocol. *Human samples miRNA isolation*. miRNAs were isolated from human FF using the miRNeasy Serum/Plasma Kit (Qiagen) according to the manufacturer’s protocol. *C57BL/6J and FVB/NJ mice ovary miRNA isolation*. miRNAs were isolated from C57BL/6J and FVB/NJ mouse ovaries that had been formalin-fixed, paraffin embedded (FFPE) using AllPrep® DNA/RNA FFPE (Qiagen). Three tissue sections of 10 µm in thickness were prepared and processed by the manufacturer’s protocol.

### cDNA synthesis and qRT-PCR for miRNA detection

qRT-PCR were performed using different protocols depending on the study. *CD1 mice ovary cDNA synthesis and qRT-PCR*.

Reverse transcription (RT) was performed using the QuantiTect Reverse Transcription Kit (Qiagen). The RT reaction was conducted using specific stem-loop primers designed for each miRNA, as previously reported^51^. Real-Time PCR was performed using Power SYBR Green Master Mix (Applied Biosystems with Applied Biosystem 7900 Real-Time PCR System). The conditions were set as previously reported in Yang et al.^51^. qRT-PCR analysis was performed in triplicate. Data obtained were normalized to the relative expression of reference gene U6 small nuclear RNA (*Rnu6*). The 2^−∆∆Ct^ method was used to calculate relative expression changes with a control group as a reference point. *Human samples cDNA synthesis and qRT-PCR*.

The same procedure, described for CD1 mice ovary cDNA synthesis and qRT-PCR was applied to human FF. Data were normalized on the relative expression of reference gene U6 small nuclear RNA (*RNU6*) transcript level. *C57BL/6J and FVB/NJ mice ovary cDNA synthesis and qRT-PCR*.

Reverse transcription (RT) was performed using the Transcriptor First Strand cDNA Synthesis Kit (Roche Diagnostics) according to the manufacturer’s instructions. The RT reaction was conducted as described above as well as Real-Time PCR, which was performed using LightCycler® 480 SYBR Green I Master (Roche Diagnostics with LightCycler® 96 System). Data were normalized as described above. qPCR primers are listed in Table S1, Supplementary information.

### Microarray experiment and bioinformatics analysis

RNA from CD1 mouse ovaries was isolated using Trizol reagent (Invitrogen) and, then, purified by RNeasy mini kit (Qiagen). Nine animals per group (Y and M) were randomly divided into three equivalent sets and a constant amount of RNA from animals in the same set was pooled into one single sample in order to eliminate individual differences within group. Three arrays were used for each group. cRNA was generated by using the Affymetrix One-Cycle Target Labelling and Control Reagent kit (AffymetrixInc), following the manufacturer’s protocol, starting from 5 μg of total RNA. The datasets obtained were analysed with GeneSpring GX 12 Software (Agilent Technologies).

### miRNA 143 and miRNA 145 predicted target gene network construction

Predicted target gene networks were constructed based on the IPA tool. A score was assigned to a network according to the fit of the original set of significant genes. This score reflects the negative logarithm of the *P* value that indicates the likelihood of the focus genes in a network being found together due to random chance. Predicted target gene network construction could not be performed for all miRNAs analysed in the study as IPA uses a large number of miRNA-related findings from the peer-reviewed literature not available for the other miRNAs.

### Accession information

Microarray data are available in the ArrayExpress database (www.ebi.ac.uk/arrayexpress) under accession nember E-MTAB-7493.

### Statistical analyses

Statistical significance between groups was determined by two-tailed Student’s *t*-test. In all cases, probability *P*-values below 0.05 were considered significant. *, **, *** indicate *P*-value < 0.05, <0.01 and <0.001, respectively. Unless otherwise indicated, at least five animals were considered for in vivo experiments. All data are presented as mean values ± standard deviation.

The strength of linear association between pairs of variables was determined by the Spearman correlation coefficient. Spearman’s rank correlation, which looks for any monotone relationship between two variables, was used to determine the relation of well-known ovarian aging parameters and miRNAs expression. The calculations were performed in R using the cor.test function, the corrplot package was used to plot the correlogram.

## Electronic supplementary material

Supplemental Material

Supplementary Table 1
