# Supplementary material for: Transcriptional landscape of mouse-aged ovaries reveals a unique set of non-coding RNAs associated with physiological and environmental ovarian dysfunctions
Source: Cell Death Discov. 2018 Dec 5;4:112. doi: 10.1038/s41420-018-0121-y (PMC6281605; doi:10.1038/s41420-018-0121-y)
Supplement: Supplementary file 1 — Supplemental Material [file 41420_2018_121_MOESM1_ESM.docx]

**Supplementary Figure 1**


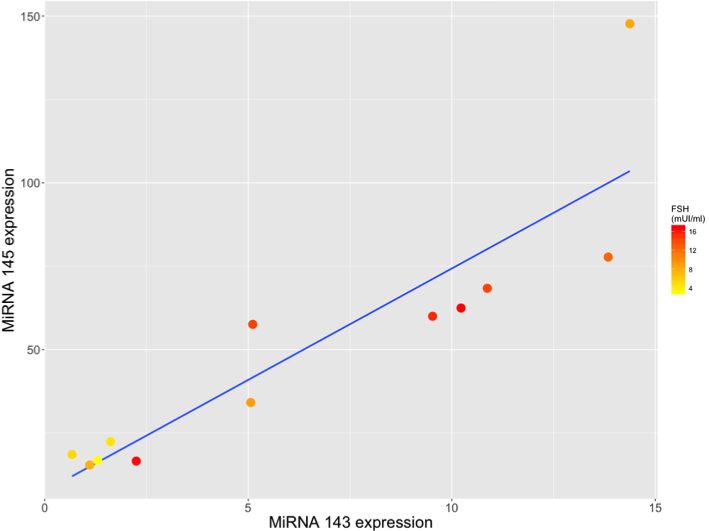


**Supplementary Figure 1. Association between *MIR143* and *MIR145* expression and serum FSH levels.** Scatterplot of relative expression show the correlation between MIR143 and MIR145 and FSH serum levels.

**Supplementary Figure 2**


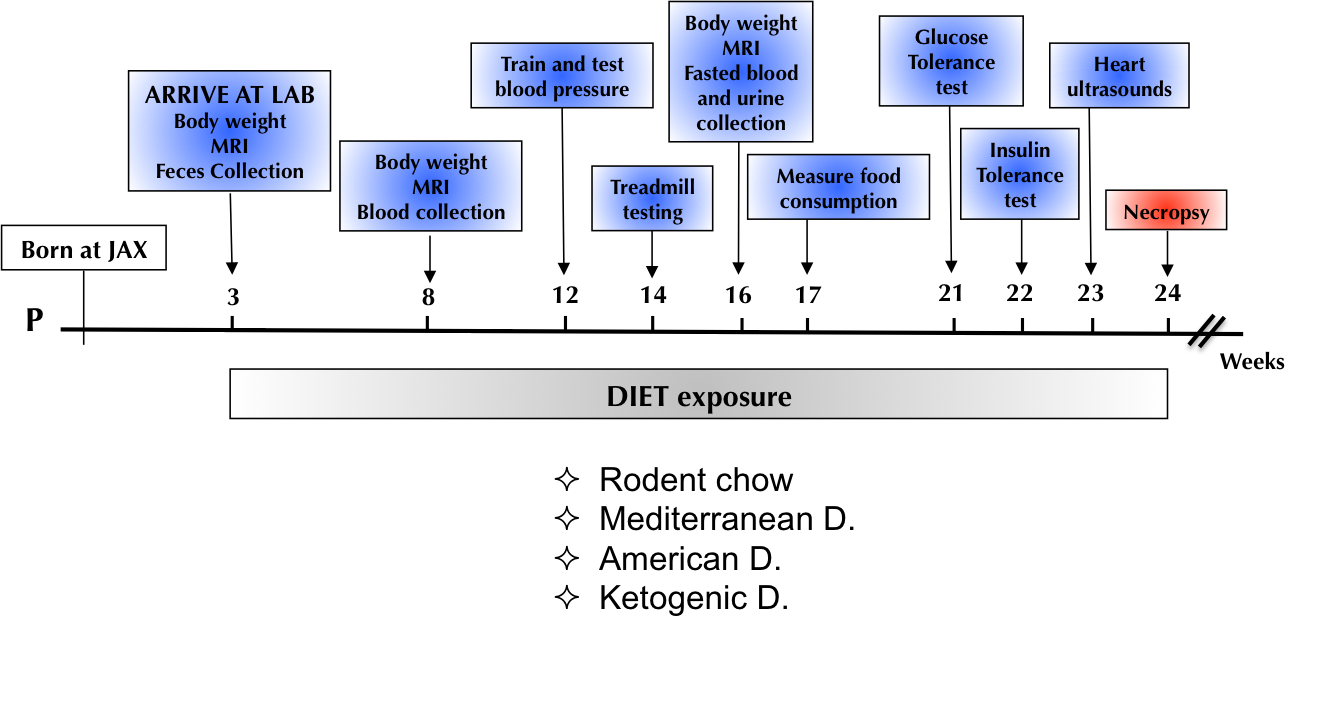


**Supplementary Figure 2. Diet experimental design.** C57BL/6J and FVB/NJ female mice were fed for 8 months starting at 6 weeks of age (*n*=5).

**Supplementary Figure 3**

**
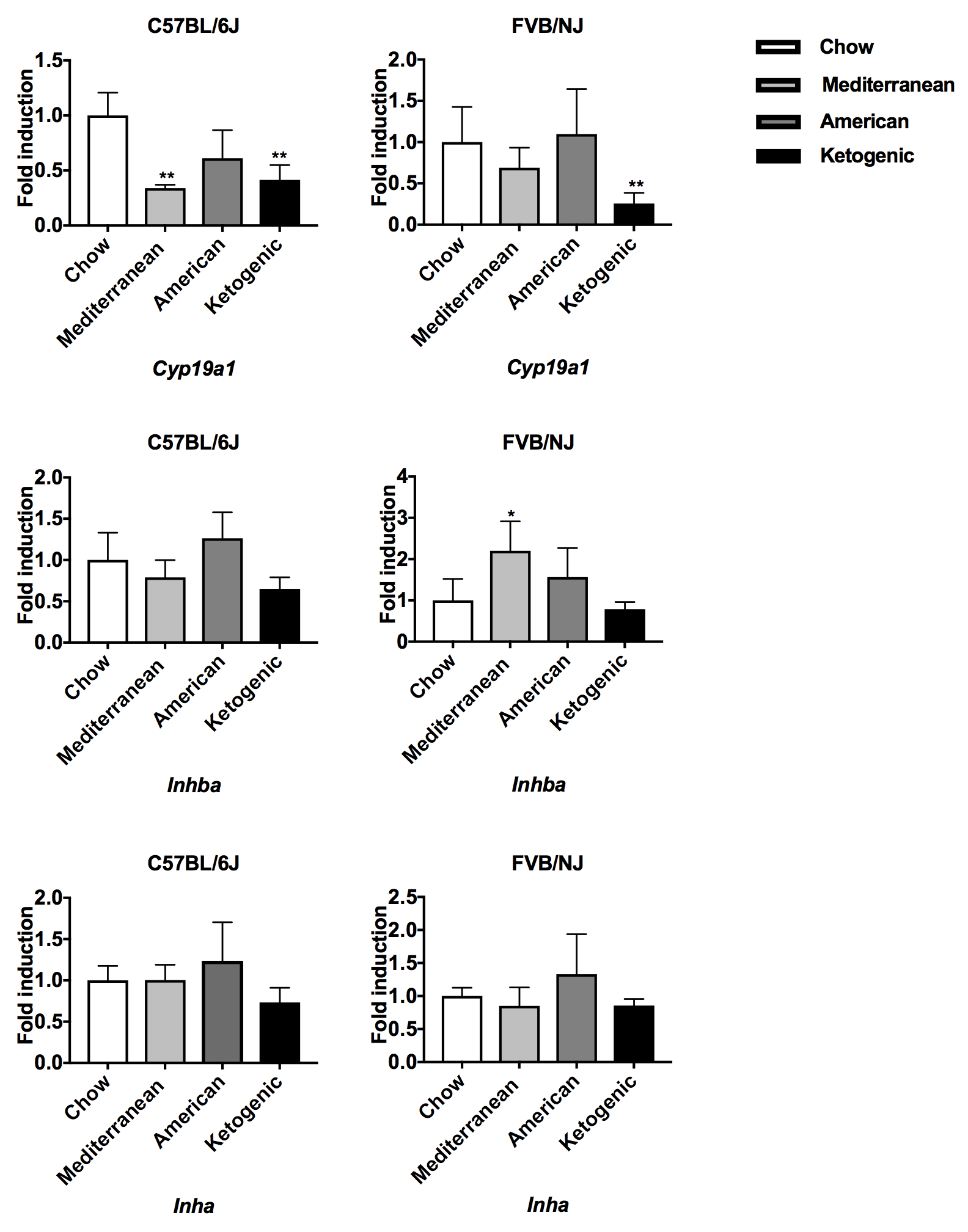
**

**Supplementary Figure 3. Markers of ovarian aging in C57BL/6J and FVB/NJ mice fed different diets.** Dietary effects on ovarian aging were determined by qRT-PCR of well-characterized ovarian aging markers in both strains.

**Supplementary Figure 4**


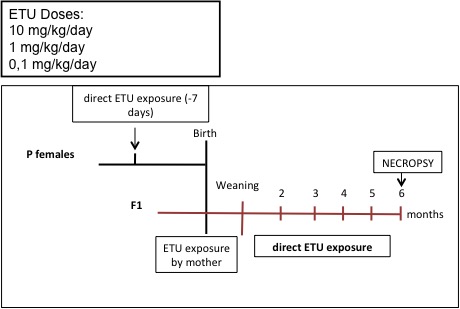


**Supplementary Figure 4. ETU experimental design.** Developmental and long-life exposure to environmentally relevant doses of ETU.

| **Markers*** | **Late reproductive period or post-menopause** |
| --- | --- |
| Cytochrome P450, family 19, subfamily a, polypeptide 1 (*Cyp19a1*) | ( ) |
| Inhibin beta-A (*Inhba*) | ( ) |
| Inhibin alpha (*Inha*) | ( ) |
| anti-Müllerian hormone (***Amh***)** | ( ) |
| Growth differentiation factor9 (*Gdf9*) | ( ) |
| Bone morphogenetic protein 15 (***Bmp15***)** | ( ) |

**Supplementary Table 2. Markers used to assess ovarian reserve.**

*Normal values are considered those found in healthy women of reproductive age

** Deregulated in microarray

**Supplementary Table 3.** **Differentially expressed genes in middle- aged ovaries included in OAGS**

| **TranscriptID** | **GeneSymbol***^A^* | **GeneDescription** | **Pathway** | **log2FC***^B^* |
| --- | --- | --- | --- | --- |
| 10468828 | *Eif3a* | Eukaryotic translation initiation factor 3, subunit A | EIF2, Regulation of eIF4 and p70S6K, mTOR (protein synthesis) | -0.84****** |
| 10485635 | *Eif3m* | Eukaryotic translation initiation factor 3, subunit M | EIF2, Regulation of eIF4 and p70S6K, mTOR (protein synthesis) | -3.49******* |
| 10566966 | *Eif4g2* | Eukaryotic translation initiation factor 4, subunit gamma | EIF2, Regulation of eIF4 and p70S6K, mTOR (protein synthesis) | -3.54******* |
| 10565802 | *Rps3* | Ribosomal protein S3 | EIF2, Regulation of eIF4 and p70S6K, mTOR (protein synthesis) | -0.90****** |
| 10435980 | *Rps24* | Ribosomal protein S24 | EIF2, Regulation of eIF4 and p70S6K, mTOR (protein synthesis) | -2.24******* |
| 10351039 | ***Gas5*** | Growth arrest specific 5 |  | -2.83******* |
| 10547073 | *Snora7a* | Small nucleolar RNA, H/ACA box 7A |  | -0.01 |
| 10586168 | ***Snord16a*** | Small nucleolar RNA, C/D box 16A |  | -2.10****** |
| 10432176 | ***Snora34*** | Small nucleolar RNA, H/ACA box 34 |  | -0.72****** |
| 10420668 | *Mir15a* | microRNA 15a | Cell Death and Survival, Protein Synthesis, Cancer, Organismal Injury and Abnormalities, Reproductive System Disease, Cell Cycle | -0.83 |

| 10442081 | *Mir99b* | microRNA 99b |  | 0.37 |
| --- | --- | --- | --- | --- |
| 10459227 | ***Mir143*** | microRNA 143 | Cell Death and Survival, Organ Morphology, Organismal Development and Function, Cancer, Organismal Injury and Abnormalities, Protein Synthesis | 1.60******* |
| 10459225 | ***Mir145*** | microRNA 145 |  | 2.18******* |
| 10400760 | ***Mir681*** | microRNA 681 |  | 1.30****** |
| 10447617 | ***Mir692-1*** | microRNA 692-1 |  | 1.53****** |

*^A^*Transcripts included in OAGS are reported in bold

*^B^*Data are reported as Log_2_ Fold change vs. Y group

**P*<0.05; ***P*<0.01; ****P*<0.001

**Supplementary Table 4 - Clinico-pathological features of patients.**

| **ID pz** | **Diagnosis** | **MII Oocytes** | **Immature**  **Oocytes** |
| --- | --- | --- | --- |
| 4 | Male infertility | 7 | 3 |
| 5 | Male infertility | 10 | 2 |
| 6 | Male infertility | 0 | 6 |
| 9 | Male infertility | 8 | 7 |
| 15 | Male infertility | 4 | 1 |
| **7** | **DOR** | **3** | **0** |
| **8** | **DOR** | **9** | **1** |
| **11** | **DOR** | **1** | **1** |
| **12** | **DOR** | **9** | **2** |
| **13** | **DOR** | **3** | **1** |
| **14** | **DOR** | **0** | **1** |
| **16** | **DOR** | **3** | **1** |
